# Países mais pobres têm mais ações pró-aleitamento materno que países ricos: estudo ecológico de 98 países

**DOI:** 10.1590/0102-311XPT007024

**Published:** 2024-11-25

**Authors:** Camila Abadia Rodrigues Meira, Catarina Machado Azeredo, Ana Elisa Madalena Rinaldi

**Affiliations:** 1 Universidade Federal de Uberlândia, Uberlândia, Brasil.; 2 Universidade Federal de Viçosa, Viçosa, Brasil.

**Keywords:** Aleitamento Materno, Produto Interno Bruto, Fatores Econômicos, Breast Feeding, Gross Domestic Product, Economic Factors, Lactancia Materna, Producto Interno Bruto, Factores Económicos

## Abstract

Buscou-se, com este estudo, verificar a associação entre valores do produto interno bruto em paridade do poder de compra (PIB PPC) e escores da ferramenta *World Breastfeeding Trends Initiative* (WBTi). Estudo ecológico realizado com 98 países de renda baixa (n = 43), média (n = 27) e alta (n = 28). A avaliação das ações pró-aleitamento materno foram obtidas da WBTi e o PIB PPC do Banco Mundial. Foram estimados média e desvio padrão (DP) das pontuações total e de cada item da WBTi. Foram usados o teste ANOVA e o teste de Tukey para comparar as médias da ferramenta WBTi segundo o PIB PPC dos países. A associação entre PIB PPC e as pontuações total e de cada item da WBTi foi analisada por regressão linear. Maiores pontuações da WBTi foram identificadas para os itens de sistemas de cuidado de saúde e nutrição (item 5: média = 6,4; DP: ±2,0) e suporte de informações válidas (item 7: média = 6,4; DP: ±2,5). As médias da pontuação total e dos itens 3 (implementação do código), 7 (suporte de informações válidas), 9 (aleitamento materno em emergências) e 10 (monitoramento e avaliação) foram superiores nos países de baixa e média renda e do item 4 (licença maternidade) nos países de alta renda (p < 0,05). Observamos associação negativa entre PIB PPC e pontuação total da ferramenta (β = -2,67; IC95%: -5,06; -0,29), item 3 (β = -0,50; IC95%: -0,91; -0,08), item 7 (β = -0,67; IC95%: -1,07; -0,27), item 8 (aleitamento materno e HIV; β = -0,59; IC95%: -1,07; -0,11) e item 9 (β = -0,91; IC95%: -1,34; -0,48). Observamos associação positiva entre PIB PPC e o item 4 (proteção à maternidade; β = 0,63; IC95%: 0,24; 1,02). Países com menor PIB PPC apresentaram maiores pontuações da ferramenta, com exceção da proteção à maternidade que apresentou maior pontuação em países com maior PIB PPC.

## Abstract (en)

This study aimed to verify the association between gross domestic product values - purchasing power parity (GDP PPP) and scores of the *World Breastfeeding Trends Initiative* (WBTi) tool. This is an ecological study carried out with 98 low-income (n = 43), middle-income (n = 27), and high-income (n = 28) countries. The evaluation of pro-breastfeeding actions was obtained from the WBTi and the GDP PPP from the World Bank. The mean and standard deviation (SD) of the total and each item WBTi scores were estimated. ANOVA and Tukey’s test were used to compare the means of the WBTi tool according to the countries’ GDP PPP. The association between GDP PPP and total and individual WBTi scores was analyzed by linear regression. Higher WBTi scores were identified for the items health and nutrition care system (item 5: mean = 6.4; SD: ±2.0) and Information Support (item 7: mean = 6.4; SD: ±2.5). The means of the total score and of items 3 (implementation of the code of marketing of breast-milk substitutes), 7 (information support), 9 (infant feeding during emergencies) and 10 (mechanisms of monitoring and evaluating systems) were higher in low- and middle-income countries, while item 4 (maternity protection in the workplace) presented a high mean in high-income countries (p < 0.05). We observed a negative association between GDP PPP and the total tool score (β = -2.67; 95%CI: -5.06; -0.29), item 3 (β = -0.50; 95%CI: -0.91; -0.08), item 7 (β = -0.67; 95%CI: -1.07; -0.27), item 8 (infant feeding and HIV; β = -0.59; 95%CI: -1.07; -0.11), and item 9 (β = -0.91; 95%CI: -1.34; -0.48). We observed a positive association between GDP PPP and item 4 (maternity protection in the workplace; β = 0.63; 95%CI: 0.24; 1.02). Countries with lower GDP PPP presented higher scores of the tool, with the exception of item 4, which had higher scores in countries with higher GDP PPP.

## Resumen

El objetivo fue verificar la asociación entre los valores del producto interno bruto, paridad del poder adquisitivo (PIB PPA) y los puntajes de la herramienta *World Breastfeeding Trends Initiative* (WBTi). Se trata de un estudio ecológico realizado con 98 países de ingresos bajos (n = 43), medios (n = 27) y altos (n = 28). La evaluación de las acciones a favor de la lactancia materna se obtuvo de la WBTi y del PIB PPP del Banco Mundial. Se estimaron la media y la desviación estándar (DE) de las puntuaciones totales y de cada ítem de la WBTi. Se utilizó ANOVA y la prueba de Tukey para comparar las medias de la herramienta WBTi según el PIB PPA de los países. La asociación entre el PIB PPA y las puntuaciones totales e individuales de los ítems WBTi se analizó mediante regresión lineal. Se identificaron puntuaciones más altas de WBTi para los ítems sistemas de cuidado de salud y nutrición (ítem 5: media = 6,4; DE: ±2,0) y soporte de información válida (ítem 7: media = 6,4; DE: ±2,5). Los promedios de la puntuación total y de los ítems 3 (implementación del código), 7 (soporte de información válida), 9 (lactancia materna en emergencias) y 10 (seguimiento y evaluación) fueron mayores en los países de ingresos bajos y medios y en el ítem 4 (licencia por maternidad) en países de altos ingresos (p < 0,05). Observamos una asociación negativa entre el PIB PPA y la puntuación total de la herramienta (β = -2,67; IC95%: -5,06; -0,29), ítem 3 (β = -0,50; IC95%: -0,91; -0,08), ítem 7 (β = -0,67; IC95%: -1,07; -0,27), ítem 8 (lactancia materna y VIH; β = -0,59; IC95%: -1,07; -0,11) y ítem 9 (β = -0,91; IC95%: -1,34; -0,48). Observamos una asociación positiva entre el PIB PPA y el ítem 4 (protección de la maternidad; β = 0,63; IC95%: 0,24; 1,02). Los países con un PIB PPA más bajo obtuvieron puntuaciones de herramientas más altas, con la excepción de la protección de la maternidad, que tuvo puntuaciones más altas en los países con un PIB PPA más alto.

## Introdução

A classificação econômica e o produto interno bruto (PIB) dos países moldam o contexto estrutural do aleitamento materno ^1^. Estudos recentes revelam uma associação inversa entre o nível de desenvolvimento econômico de um país e as taxas de aleitamento materno ^2^ ^,^ ^3^. Nos países de alta renda, embora as taxas de aleitamento materno sejam baixas, houve tendência de aumento entre 1990 e 2015 ^2^. Adicionalmente, países com a mesma classificação econômica apresentam trajetórias distintas de aleitamento materno, enquanto alguns países apresentam aumento em suas taxas, outros países apresentam queda ^2^ ^,^ ^3^ ^,^ ^4^.

As taxas de aleitamento materno podem aumentar quando os países implementam e coordenam duas ou mais ações e políticas pró-aleitamento materno ^1^ ^,^ ^2^ ^,^ ^5^. Em países de baixa renda, há predomínio de ações que visam realizar o treinamento de profissionais de saúde, uso estratégico de dados e meios de comunicação em massa ^2^. Nos países de renda média, as principais ações pró-aleitamento materno foram o treinamento dos profissionais de saúde, a implementação de hospitais amigos da criança, o fortalecimento da proteção à maternidade e a implementação do *Código Internacional de Comercialização de Substitutos do Leite Materno* ^2^. Já nos países de alta renda, as principais ações foram legislações de proteção do aleitamento materno em locais públicos, cobertura de seguro obrigatório para aconselhamento de lactação e bombas de leite materno e para fornecimento de espaço e tempo para a ordenha do leite materno no trabalho ^2^. Adicionalmente, destaca-se o forte envolvimento e a participação da sociedade civil para fortalecer o aleitamento materno como elemento potente em todos os países, independentemente da classificação econômica ^1^ ^,^ ^2^ ^,^ ^5^.

A ferramenta *World Breastfeeding Trends Initiative* - WBTi (tradução livre dos autores: Iniciativa Mundial sobre Tendências da Amamentação) - permite a análise do panorama nacional e mundial de políticas, programas e ações pró-aleitamento materno, desde a sua criação em 2004 ^6^ ^,^ ^7^. Os objetivos principais da WBTi são monitorar e avaliar o progresso das políticas, programas e financiamentos pró-aleitamento materno para embasar a tomada de decisões sobre o tema nos países ^8^. Em estudo realizado com dados dessa ferramenta em 40 países de baixa e média renda, foi verificado que nos últimos 20 anos a pontuação total para a maioria dos países foi inferior a 70 pontos de um total de 100 pontos, com desempenho pior para os itens que se referem às ações em situação de emergência, HIV e alimentação infantil e de proteção à maternidade ^9^.

Atualmente, os estudos disponíveis na literatura com os dados da ferramenta WBTi se concentram em descrever seu status, sem verificar sua associação com às informações de nível econômico dos países ^6^ ^,^ ^9^ ^,^ ^10^ ^,^ ^11^ ^,^ ^12^ ^,^ ^13^, especialmente nos países de alta renda. Adicionalmente, as informações sobre quais os tipos de ações predominantes em cada país segundo a classificação econômica são escassos. A utilização da ferramenta WBTi pode gerar avanços na implementação das políticas e dos programas pró-aleitamento materno nos países ^6^ ^,^ ^9^ ^,^ ^10^ ^,^ ^11^ ^,^ ^12^ ^,^ ^13^. Sendo assim, o objetivo deste estudo foi verificar a associação entre os valores do PIB *per capita* e os escores da ferramenta WBTi.

## Métodos

### Desenho do estudo, fonte dos dados e critérios de inclusão

Este é um estudo ecológico realizado com dados de 98 países de baixa, média e alta renda. Os dados de políticas e programas pró-aleitamento materno nos países foram provenientes da ferramenta WBTi (https://www.worldbreastfeedingtrends.org/) e a classificação de renda dos países foram obtidos pelo *website* do Banco Mundial (https://www.worldbank.org/en/home). Foram incluídos todos os países que realizaram pelo menos uma avaliação da ferramenta WBTi e foi selecionado o ano mais recente da avaliação, quando os países tinham mais de uma avaliação WBTi.

### Preditor

A classificação de renda dos países foi realizada a partir dos valores do PIB em poder de paridade de compra (PIB PPC) cujos pontos de corte são propostos pelo Banco Mundial, correspondente ao ano de avaliação da ferramenta WBTi para cada país do estudo. Dessa forma, os países foram classificados em três faixas: baixa renda para valor do PIB PPC inferior a USD 4.255; média renda para valores do PIB PPC entre USD 4.256 e USD 13.205; e alta renda para valores superiores a USD 13.205. Com base nessa classificação, 43 países analisados eram classificados como de baixa renda (43,9%), 27 de média renda (27,6%) e 28 de alta renda (28,6%).

### Desfechos

Os desfechos foram as pontuações total e de cada item da ferramenta WBTi. A ferramenta WBTi é composta por 10 itens relacionados às políticas e programas pró-aleitamento materno, os quais são: política nacional, governança e financiamento (item 1); iniciativa Hospital Amigo da Criança/10 passos para o sucesso do aleitamento materno (item 2); implementação do *Código Internacional de Comercialização de Substitutos do Leite Materno* (item 3); proteção à maternidade (item 4); sistemas de cuidado de saúde e nutrição (item 5); serviços de aconselhamento para grávidas e lactantes (item 6); suporte de informações válidas (item 7); alimentação infantil e HIV (item 8); alimentação de bebês e crianças pequenas em emergências (item 9); monitoramento e avaliação (item 10). Cada item relacionado às políticas e aos programas pró-aleitamento materno recebe uma pontuação que varia de 0 a 10 e é composto por uma lista de subitens para que a pontuação possa ser realizada. Esses subitens mensuram a presença de uma determinada política, sua implementação e, em alguns itens, um indicador de sua efetividade. Apresentamos um exemplo da composição do primeiro indicador, Item 1 - política nacional, programa e coordenação: esse indicador é composto por oito itens que indicam de forma geral a presença de uma política de aleitamento materno oficialmente adotada/aprovada pelo governo (sim/não); presença de um plano nacional e se é financiado; presença de um comitê nacional e como é coordenado. A soma dos 10 itens compõem a pontuação total, com variação de 0 a 100. Pontuações acima de 70 pontos para esses itens representam maiores avanços na existência e na implementação das políticas e dos programas pró-aleitamento materno.

### Análise estatística

Primeiramente, foi realizado o levantamento das pontuações total e de cada item da ferramenta WBTi, do PIB PPC e a respectiva classificação de renda (faixas) dos países selecionados para o estudo. Posteriormente, foram estimados a média e o desvio-padrão das pontuações total e de cada item da ferramenta WBTi segundo a classificação econômica do país.

O teste de análise de variância (ANOVA) e o teste de Tukey foram realizados para comparar as médias das pontuações da ferramenta (total e de cada item) segundo a classificação de renda (baixa, média e alta renda). O valor de p < 0,05 foi adotado para significância.

Inicialmente, foi feito o diagrama de dispersão entre os valores do PIB PPC (*log*) e a pontuação do WBTi (pontuações parciais e total). Posteriormente, foi realizada regressão linear para verificar a associação entre os valores do PIB PPC (em base logarítmica) e as pontuações total e de cada item da ferramenta WBTi.

Todas as análises foram realizadas usando o STATA SE, versão 15.1 (https://www.stata.com). Não foi necessária submissão da pesquisa a comitê de ética em pesquisa, visto que todos os dados da ferramenta WBTi e PIB e classificação econômica dos países são de domínio público e gratuito.

## Resultados

A classificação da pontuação total da ferramenta WBTi variou de 19 pontos na Líbia a 87,5 pontos em Cuba. Somente 14 países apresentaram pontuação acima de 70, sendo sete de baixa renda, seis de média renda e somente um país de alta renda. Em países de baixa renda, a pontuação variou de 22,5 pontos a 77 pontos; em países de média renda, variou de 19 pontos a 87,5 pontos; e, em países de alta renda, variou de 25,5 pontos a 74,5 pontos (dados não mostrados em tabelas).

Na Tabela 1, estão as médias da pontuação total e de cada item da ferramenta WBTi. A média da pontuação total foi de 53,6 pontos (±14,5). As maiores médias das pontuações parciais foram observadas nos itens 5 (6,4±2,0) e 7 (6,4; ±2,5), seguidos do item 3 com média de 6,1 (±2,4).

**Tabela 1:** Média e desvio padrão (DP) das pontuações parciais e total da ferramenta *World Breastfeeding Trends Initiative* (WBTi) segundo classificação de renda do país.

	Total	Itens									
		1	2	3	4	5	6	7	8	9	10
	Média (DP)	Média (DP)	Média (DP)	Média (DP)	Média (DP)	Média (DP)	Média (DP)	Média (DP)	Média (DP)	Média (DP)	Média (DP)
Países	53,6 (±14,5)	5,8 (±2,8)	4,8 (±2,6)	6,1 (±2,4)	5,1 (±2,3)	6,4 (±2,0)	5,7 (±2,0)	6,4 (±2,5)	5,5 (±2,9)	2,3 (±2,8)	5,6 (±2,8)
Classificação de renda											
Baixa	56,5 (±14,5)^a^	6,0 (±2,4)^a^	4,3 (±2,8)^a^	6,4 (±2,6)^a^	4,4 (±2,1)^a^	6,8 (±2,2)^a^	6,0 (±1,9)^a^	7,3 (±2,1)^a^	6,0 (±3,1)^a^	3,2 (±3,1)^a^	6,1 (±2,7)^a^
Média	54,4 (±15,6)^a,b^	6,3 (±2,7)^a^	5,6 (±2,5)^a^	6,6 (±2,3)^a^	5,4 (±2,4)^b^	6,3 (±1,9)^a^	5,1 (±2,4)^a^	5,7 (±2,5)^b^	5,5 (±2,3)^a^	2,5 (±2,3)^b^	5,4 (±2,7)^a,b^
Alta	48,4 (±12,3)^b^	4,8 (±3,4)^a^	4,9 (±2,3)^a^	5,3 (±2,1)^b^	5,9 (±2,4)^b^	5,9 (±1,8)^a^	5,9 (±5,9)^a^	5,8 (±5,7)^b^	4,8 (±3,1)^a^	0,8 (±1,4)^c^	5,1 (±2,9)^b^

Nota: item 1 - política nacional, governança e financiamento; item 2 - iniciativa Hospital Amigo da Criança/10 passos para o sucesso do aleitamento materno; item 3 - implementação do *Código Internacional de Comercialização de Substitutos do Leite Materno*; item 4 - proteção à maternidade; item 5 - sistemas de cuidado de saúde e nutrição; item 6 - serviços de aconselhamento para grávidas e lactantes; item 7 - suporte de informações válidas; item 8 - alimentação infantil e HIV; item 9 - alimentação de bebês e crianças pequenas em emergências; item 10 - monitoramento e avaliação. Letras diferentes indicam diferenças significativas segundo classificação de renda (valor de p < 0,05).

Destacamos que a maioria das pontuações de cada item se enquadraram entre 4,3 e 7,3 pontos e que as piores pontuações foram vistas no item alimentação de bebês e crianças pequenas em emergências (item 9) em todas as classificações de renda (baixa = 3,2±3,1; média = 2,5±2,3; alta = 0,8±1,4) (Tabela 1). De forma geral, as médias das pontuações total e de cada item foram maiores para a maioria dos itens da ferramenta nos países de baixa e média renda em comparação com os países de alta renda. Observamos média inferior do item 3 para os países de alta renda em aos de média e baixa renda (p < 0,05). Verificamos média superior do item 7 para países de baixa renda em comparação aos de média e alta renda (p < 0,05). Observamos médias superiores nos países de baixa renda em comparação aos de alta renda para a pontuação total e o item 10 (p < 0,05). Observamos média inferior do item 9 para países de alta renda em comparação aos de média e baixa renda (p < 0,05). Ainda sobre esse item, observamos média inferior em países de média renda em comparação a países de baixa renda (p < 0,05). Já para o item 4 (licença maternidade), observamos a média superior nos países de alta renda e média renda em comparação com os de baixa renda (p < 0,05) (Tabela 1).

**Figura 1:**
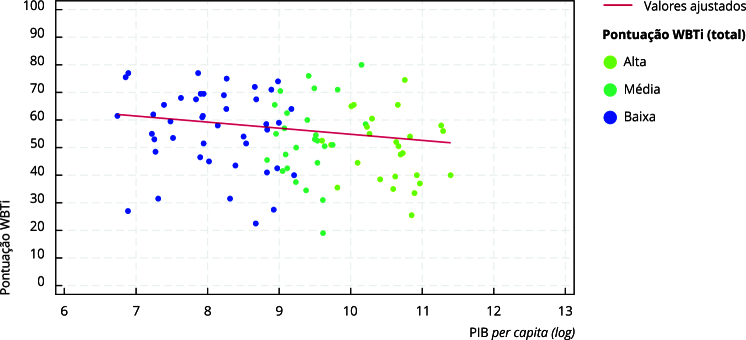
Diagrama de dispersão entre o produto interno bruto (PIB) *per capita* (*log*) e a pontuação total da ferramenta *World Breastfeeding Trends Initiative* (WBTi).

Observamos associação negativa entre o valores do PIB *per capita* e a pontuação total da ferramenta WBTi (β = -2,67; IC95%: -5,06; -0,29) (Figura 1). Ainda, notamos associação negativa para os itens implementação do Código (item 3) (β = -0,50; IC95%: -0,91; -0,08), suporte de informações válidas (item 7) (β = -0,67; IC95%: -1,07; -0,27), alimentação infantil e HIV (item 8) (β = -0,59; IC95%: -1,07; -0,11) e alimentação de bebês e crianças pequenas em emergências (item 9) (β = -0,91; IC95%: -1,34; -0,48). Observamos também associação positiva entre o valor do PIB *per capita* e a pontuação do item proteção à maternidade (item 4) (β = 0,63; IC95%: 0,24; 1,02).

## Discussão

Verificamos médias superiores da pontuação total e de quatro itens da ferramenta (implementação do código, o suporte de informações válidas, as políticas sobre aleitamento materno e HIV e o aleitamento materno em emergências) nos países de baixa e média renda em relação aos países de alta renda, indicando que mais ações pró-aleitamento materno são implementadas nesses países.

Com relação ao item 3, o conteúdo analisado se refere ao grau de sua implementação e aplicação das resoluções da Assembleia Mundial de Saúde por medidas legais e de fiscalização da aplicação do mesmo ^9^. A presença de um código mais robusto nos países de baixa e de média renda busca coibir a influência das empresas de substitutos do leite materno nesses países. As indústrias de fórmulas infantis têm sua sede nos países de alta renda, nos quais o mercado consumidor já está praticamente saturado. Há, por parte das indústrias de fórmulas, pressão governamental para o enfraquecimento do código ^2^ ^,^ ^5^ ^,^ ^14^ ^,^ ^15^. Isso pode influenciar nas pontuações menores da ferramenta WBTi e mostra a fragilidade das políticas em função da presença das empresas de fórmula. Uma estratégia para expandir os lucros e o mercado consumidor é a venda dos produtos nos países de baixa e média renda, juntamente com táticas de marketing agressivas. Assim, nesses países há menor pressão governamental para coibir o código, possibilitando medidas mais robustas de controle do marketing agressivo dessas empresas ^2^ ^,^ ^5^ ^,^ ^14^ ^,^ ^15^. Outra possível situação seria a existência do código nos países de baixa e média renda prévia à entrada em massa das empresas de fórmula infantil. Embora a maioria dos países reconheça a importância do código por meio de legislações que proíbam promoções de substitutos do leite materno, as principais barreiras encontradas nos países com relação à sua legislação incluem a ausência de vontade política, a interferência da indústria de substitutos do leite materno, a compreensão limitada do código, os recursos humanos e financeiros insuficientes e a ausência de responsabilização, monitoramento e mecanismos de aplicação do código ^15^.

Destacamos que 194 países são membros da Organização Mundial da Saúde (OMS) e 144 (74%) países adotaram alguma disposição legal para implementar o código ^15^. Somente 16% das nações adotaram medidas que estão significativamente em consonância com o código, e apenas 15% têm leis que abrangem integralmente seu escopo. Isso significa que, para a maioria dos países, o código ainda não foi integralmente incorporado à legislação ^15^. Outro ponto importante é que o potencial aumento do PIB de um país pode gerar maior abertura para o crescimento de consumo de fórmulas ^3^ e maior interesse das indústrias em mercados emergentes, especialmente em países de baixa renda. A literatura mostra que o consumo de dietas com níveis mais elevados de fórmula infantil acelerou nas últimas décadas, especialmente em países altamente populosos de renda média-baixa e de média-alta ^5^. Adicionalmente, cerca de um em cada três neonatos em países de baixa e média renda recebe alimentação pré-láctea, e apenas um em cada dois neonatos é colocado no seio materno na primeira hora de vida ^1^. Por outro lado, vários países, principalmente os de alta renda, não dispõem de dados sobre aleitamento materno, indicando pouco valor dado ao tema ^2^ ^,^ ^14^. Ainda, a ideia de que a amamentação é antitrabalho e antifeminista é disseminada em meios de comunicação nesses países ^16^.

Mesmo os países com boas pontuações da ferramenta WBTi precisam fazer esforços para implementar e monitorar de forma efetiva o código, pois apenas a presença de uma lei não é suficiente para provocar mudanças ^6^ ^,^ ^9^. Espera-se que uma forte defesa global e a crescente disponibilidade de ferramentas para implementar o código superarão muitas dessas barreiras e acelerarão o progresso em seu cumprimento integral ^15^. A implementação integral do código possibilitaria melhores decisões das mães e famílias sobre a alimentação de bebês e crianças pequenas, por meio de informações livres de influências comerciais e práticas de marketing enganosas, e deveria ser vista pelos países como uma prioridade de saúde pública e de direitos humanos ^15^.

Outro item da ferramenta WBTi com maior pontuação em países de baixa renda foi o item suporte de informações válidas (item 7). Esse item examina o tipo de informação, se é tecnicamente correta ou não, e quais estratégias de informação, educação e comunicação são usadas sobre alimentação de bebês e crianças pequenas pelos Estados ^6^ ^,^ ^9^. Embora países mais pobres sejam mais vulneráveis, eles apresentam uma preocupação maior em relação ao aleitamento materno e incluem o manejo e orientações sobre aleitamento materno nos serviços de saúde, influenciando no desenvolvimento de mais políticas e programas, o que poderia justificar, ao menos em parte, nossos resultados ^1^. Destacamos também que países mais pobres com normas e tradições de priorização do aleitamento materno podem ter mais facilidade de estruturar políticas e programas mais fortes com ações de promoção e de educação em saúde na rotina dos serviços e incorporar mais indicadores relacionados à alimentação infantil ^1^ ^,^ ^12^.

Do total de países analisados neste estudo, 24,5% são países da região da África Subsaariana, considerada uma das regiões mais pobres do mundo. Apesar do atual panorama econômico dessa região, o item políticas sobre alimentação infantil e HIV (item 8) da ferramenta WBTi foi melhor pontuado nos países de baixa renda. Esse item examina que tipo de apoio na alimentação de bebês e crianças pequenas é disponibilizado via políticas e programas às mulheres soropositivas ^9^. Países mais pobres são mais vulneráveis à infecção pelo HIV, apresentando prevalência extremamente elevada, e possuem maior preocupação em medidas para conter a infecção pelo vírus, justificando os resultados encontrados. Em países de alta renda, a prevalência de HIV não é tão alta, o sistema de saúde é mais estruturado e tem melhores condições de saúde na prevenção e no tratamento do HIV.

Notamos que alimentação de bebês e crianças pequenas em emergências (item 9) apresentou maior pontuação nos países de baixa renda. Tal item é importante para identificar quais políticas e programas existem nos países a fim de proteger e dar apoio às mães na alimentação adequada de seus bebês durante desastres ^6^ ^,^ ^9^. Emergências e desastres, especialmente guerras civis e emergências climáticas, são mais frequentes nos países de baixa renda. Dessa forma, esses países teriam maiores preocupações e, consequentemente, mais políticas sobre esse tema. Em contrapartida, países mais ricos conseguem se estruturar mais rapidamente quando acontecem desastres, como terremotos, e têm melhores condições de saúde, então não teriam maiores preocupações. Um sistema de saúde mais estruturado em países mais ricos poderia influenciar na resposta às emergências.

O tipo de estrutura no sistema de saúde necessária para promover mais o aleitamento materno em emergências poderia ser: treinamento específico de profissionais de saúde para situações de emergência; planos de contingência com estratégias para apoio ao aleitamento materno; estoque de suprimentos como *kits* de apoio à aleitamento materno e suplementos nutricionais para lactantes; espaços seguros e privados em abrigos e centros de emergência para apoio ao aleitamento materno; unidades móveis de saúde equipadas para apoiar famílias em áreas afetadas; campanhas de conscientização e grupos de apoio à aleitamento materno; sistemas de monitoramento para rastrear taxas de aleitamento materno durante e após situações de emergência; coleta e análise de dados para avaliar eficácia das intervenções pró-aleitamento materno e identificar áreas de melhoria; políticas de suporte como proteção à maternidade para garantir direito ao aleitamento materno em qualquer circunstância; implementação e cumprimento do código para evitar promoção indevida de fórmulas; colaboração intersetorial entre diferentes setores (saúde, assistência social, defesa civil); e parcerias com organizações internacionais para fornecimento de apoio técnico e logístico ^17^ ^,^ ^18^ ^,^ ^19^. O item alimentação infantil de bebês e crianças pequenas em emergências (item 9) precisa ter preferência no tocante às políticas e aos programas pró-aleitamento materno ^6^ ^,^ ^9^ e a literatura ainda nos mostra que a maioria dos países falha no preparo do suporte às mulheres para alimentar seus bebês de maneira ideal durante desastres ^6^ ^,^ ^9^ ^,^ ^12^ ^,^ ^13^.

Neste estudo, países de alta renda tiveram melhor pontuação do item proteção à maternidade (item 4). Esse item é usado para medir a situação dos direitos à maternidade, incluindo licença remunerada, intervalos para amamentação, licença paternidade, acomodação no local de trabalho para amamentação ou extração de leite materno, creches ou instalação de cuidado da criança e sistemas de monitoramento sobre direitos de maternidade ^6^ ^,^ ^9^. A maioria dos países de alta renda incluídos neste estudo valorizaram mais a proteção à maternidade do que países mais pobres. As possíveis razões para isso podem ser as melhores estruturas de empregabilidade e o maior percentual de mulheres com trabalho formal, questões relativas ao trabalho mais estruturadas, arcabouço social e econômico mais estruturado e organizado. Além disso, é importante ressaltar que a diminuição da taxa de natalidade em países ricos, juntamente com iniciativas para promover a parentalidade, como a ampliação das licenças maternidade e paternidade, também podem ser razões para a valorização da proteção à maternidade nesses países ^20^. Países com pontuação baixa da licença maternidade precisam de implementação e fiscalização das políticas de bem-estar social, pró-aleitamento materno e previdência.

Fatores contextuais, como o PIB *per capita*, fazem parte dos determinantes da amamentação ^1^ ^,^ ^2^ ^,^ ^3^. Durante o século XX, a aleitamento materno foi menos frequente em países de alta renda e, no século XXI, foi menos comum em países de baixa e média renda em mulheres com maiores renda, escolaridade e que residem na área urbana ^2^. Essa tendência pode ser explicada pela percepção de que os substitutos do leite materno eram considerados modernos e prestigiados, enquanto o aleitamento materno muitas vezes era associado à pobreza e à falta de sofisticação ^2^. Mesmo após mais de um quarto de século da implementação de várias políticas e programas pró-aleitamento materno, como a *Declaração de Innocenti* e a iniciativa Hospital Amigo da Criança, as taxas globais de aleitamento materno ainda estão consideravelmente aquém das metas internacionais ^2^. É crucial avaliar os investimentos destinados a promover o aleitamento materno, tanto em contextos de maior poder aquisitivo quanto em regiões mais pobres, considerando os custos associados à não promoção dessa prática ^2^. Nossos resultados corroboram a literatura e mostraram que todos os itens da ferramenta em todos os níveis de renda estão aquém do desejado (9,1 pontos da ferramenta), especialmente nos países de alta renda.

Atualmente, 98 países realizaram pelo menos uma aplicação da ferramenta WBTi e encontramos no nosso estudo uma variação de 68,5 pontos da pontuação total da ferramenta entre esses países. Há também discrepâncias do desempenho dos países na ferramenta dentro de uma mesma região geográfica, conforme estudo prévio realizado com cinco países da América Latina ^21^. Essa grande variabilidade da pontuação da ferramenta WBTi mostra a grande heterogeneidade global do desenvolvimento, da implementação e da avaliação das ações pró-aleitamento materno entre os países. O processo de avaliação do WBTi é baseado em critérios objetivos, e cada país tem uma equipe treinada para realizar a avaliação. Adicionalmente, para a aplicação da ferramenta é necessária a mobilização de uma equipe no nível nacional.

Destacamos como principal ponto positivo deste estudo a análise da ferramenta WBTi e sua associação com o PIB *per capita* dos países, ausente nos estudos anteriores ^6^ ^,^ ^9^ ^,^ ^10^ ^,^ ^11^ ^,^ ^12^ ^,^ ^13^. Adicionalmente, podemos identificar a necessidade de avanços na implementação das ações pró-aleitamento materno, especialmente nos países de alta renda.

Entretanto, a limitação principal deste estudo é inerente à ferramenta WBTi: a presença de países com pontuações de cada item e da pontuação total não refletir os mesmos quesitos pontuados, já que as pontuações dos 10 itens dos programas e políticas pró-aleitamento materno são geradas a partir da pontuação dos subitens de cada item. Os subitens são variados e, consequentemente, os panoramas dos países também. Ou seja, tendo em vista que existe a variabilidade dos subitens dos 10 itens, a ferramenta não consegue distinguir o *set* de um país para outro país somente pela pontuação parcial ou total da ferramenta.

O aleitamento materno é moldado por uma série de fatores históricos, socioeconômicos, culturais e individuais. Países de baixa renda possuem determinantes contextuais de aleitamento materno, como o nível econômico investigado nesse estudo, que influenciam a investir mais em ações sobre a implementação do código, suporte de informações válidas, políticas sobre aleitamento materno e HIV e aleitamento materno em emergências. Países de alta renda investem mais em ações para a proteção à maternidade. Esforços de monitoramento e avaliação das ações pró-aleitamento materno por meio da ferramenta WBTi ampliarão a probabilidade de adoção materna ou familiar do aleitamento materno. A sinergia gerada pela combinação de diferentes ações pró-aleitamento materno e a participação da sociedade civil nos países são elementos importantes que também auxiliam no aumento das taxas de aleitamento materno. Faz-se necessário dispor de esforços para melhorar os itens que receberam baixas pontuações no WBTi e aprimorar aqueles com maiores pontuações para que os avanços sejam mantidos.
